# Insights on the biochemical and cellular changes induced by heat stress in the *Cladocopium* isolated from coral *Mussismilia braziliensis*

**DOI:** 10.3389/fmicb.2022.973980

**Published:** 2022-10-10

**Authors:** Michele S. Lima, Lidilhone Hamerski, Tatiana A. Silva, Maria Luíza R. da Cruz, Tooba Varasteh, Diogo A. Tschoeke, Georgia C. Atella, Wanderley de Souza, Fabiano L. Thompson, Cristiane C. Thompson

**Affiliations:** ^1^Laboratory of Microbiology, Biology Institute, Federal University of Rio de Janeiro (UFRJ), Rio de Janeiro, Brazil; ^2^Walter Mors Institute of Research on Natural Products, Federal University of Rio de Janeiro (UFRJ), Rio de Janeiro, Brazil; ^3^Laboratory of Celullar Ultrastructure Hertha Meyer, Biophysics Institute Carlos Chagas Filho, Federal University of Rio de Janeiro (UFRJ), Rio de Janeiro, Brazil; ^4^National Center for Structural Biology and Bioimaging (Cenabio), Federal University of Rio de Janeiro (UFRJ), Rio de Janeiro, Brazil; ^5^Biomedical Engineering Program – COPPE, Federal University of Rio de Janeiro (UFRJ), Rio de Janeiro, Brazil; ^6^Laboratory of Lipids Biochemistry and Lipoprotein, Biochemistry Institute Leopoldo de Meis, Federal University of Rio de Janeiro (UFRJ), Rio de Janeiro, Brazil; ^7^Center of Technology-CT2, SAGE-COPPE, Federal University of Rio de Janeiro (UFRJ), Rio de Janeiro, Brazil

**Keywords:** *Mussismilia braziliensis*, *Cladocopium*, metabolism, electronic microscopy, lipidomics, heat stress biomarkers

## Abstract

Corals are treatened by global warming. Bleaching is one immediate effect of global warming, resulting from the loss of photosynthetic endosymbiont dinoflagellates. Understanding host-symbiont associations are critical for assessing coral’s habitat requirements and its response to environmental changes. *Cladocopium* (formerly family Symbiodiniaceae clade C) are dominant endosymbionts in the reef-building coral, *Mussismilia braziliensis*. This study aimed to investigate the effect of temperature on the biochemical and cellular features of *Cladocopium*. Heat stress increased oxygen (O_2_) and decreased proteins, pigments (Chla + Chlc2), hexadecanoic acid- methyl ester, methyl stearate, and octadecenoic acid (Z)- methyl ester molecules. In addition, there was an increase in neutral lipids such as esterified cholesterol and a decrease in free fatty acids that may have been incorporated for the production of lipid droplets. Transmission electron microscopy (TEM) demonstrated that *Cladocopium* cells subjected to heat stress had thinner cell walls, deformation of chloroplasts, and increased lipid droplets after 3 days at 28°C. These findings indicate that thermal stress negatively affects isolated *Cladocopium* spp. from *Mussismilia* host coral.

## Introduction

Corals are under threat due to ocean warming, pollution, and acidification (Hoegh-Guldberg et al., 2007; Reichert et al., 2021). The recent Intergovernmental Panel on Climate Change (IPCC) report suggests an increase of at least 1.5°C which will further affect corals. According to IPCC by 2040, there will be an increase in the average temperature of the planet by at least 1.5°C (Masson-Delmotte, et al., 2018). Coral-dominated reef communities will become rare until the mid-21st century (Hoegh-Guldberg and Bruno, 2010). Dinoflagellates of the family Symbiodiniaceae are globally important in marine ecosystems. The phylogenetic groupings commonly referred to as “clades” are now recognized as different genera (LaJeunesse et al., 2018). Currently, about half of the total number of species are given formal names and have been revised and divided into seven genera (LaJeunesse et al., 2018). These genera are relevant cnidarian endosymbionts (Suggett et al., 2017). Dinoflagellate endosymbionts can live inside coral tissues at extremely high densities (>10^6^ cells/cm^−2^; Muller-Parker and Davy, 2005). The most common genera in symbiosis with corals are *Cladocopium* (former Clade C) and *Symbiodinium* (former Clade A; LaJeunesse et al., 2018). However, global and local environmental disturbances may cause losses of symbionts from the hosts (coral bleaching), with potential coral mortality (Jokiel and Brown, 2004; Baker et al., 2008).

The most notorious effects of increased seawater temperature are bleaching and coral disease (Ruiz-Moreno et al., 2012). Some species of dinoflagellates are endosymbionts of marine animals playing an important role in coral reefs but also contributing to carbon fixation (Janouškovec et al., 2017). Coral colonies have the potential to adapt to changing physical conditions by their ability to acquire different Symbiodiniaceae types, including temperature-resistant strains (Baker, 2003). In addition, the ability of lineages of *Symbiodinium* to efficiently synthesize photo protectors as mycosporine-like amino acids (Silva-Lima et al., 2015) seems to allow them to occupy shallow areas associated with high temperatures (Picciani et al., 2016), while *Cladocopium* may be correlated with high seawater turbidity (Picciani et al., 2016). *Cladocopium* may confer resistance to bleaching in scleractinians because of its heat tolerance (Varasteh et al., 2018). *Cladocopium* is physiologically diverse, with clades adapted to a wide range of temperatures and irradiances (LaJeunesse et al., 2018).

High temperature induces reduction of cells, accumulation of lipid droplets, and disorganization of subcellular organelles in the endosymbiont (Fujise et al., 2014). The formation of different lipid droplets was observed in Symbiodiniaceae when cultured in temperatures of 15 and 30°C (Pasaribu et al., 2016). Endosymbionts exposed to elevated temperatures also show signs of death as necrosis and apoptosis after 1-day exposure to heat stress (Sammarco and Strychar, 2013). However, these previous studies focused on a restricted number of endosymbionts mainly from the Pacific Ocean, and the Caribbean (Jiang et al., 2014; Nitschke et al., 2014; Weng et al., 2014; Ayalon et al., 2021).

The South Atlantic Ocean has proportionally more generalist coral species associated with Symbiodiniceae in comparison to Indo-Pacific and Caribbean (Mies et al., 2020; Varasteh et al., 2021). For instance, *Cladocopium* associated with *Madracis* was found in Abrolhos Bank in the southwestern Atlantic among other Symbiodiniaceae types (Varasteh et al., 2021). Furthermore, *Mussismilia* corals from Abrolhos are dominated by *Cladocopium* (Silva-Lima et al., 2015). Water temperature in this region varies between 25 and 28°C (Reis et al., 2016; Silva-Lima et al., 2021). The coral provides nitrogen metabolites and CO_2_ for the endosymbionts, meanwhile, the symbiont provides O_2_ and essential nutrients for the coral (Hillyer et al., 2016). Over 95% of nutrients obtained by the coral host come from the symbiont, in the form of lipids, sugars, proteins, and carbohydrates (Stanley, 2006; Antonelli et al., 2015). This symbiosis accelerates coral growth rates and favors reef formation. However, severe bleaching events have occurred globally recently (Hughes et al., 2017). *Mussismilia* corals appear to be less susceptible to bleaching than other corals, as a possible effect of oceanographic parameters and cellular features of the coral holobiont (Lisboa et al., 2018; Mies et al., 2020). However, it is unclear how the *Mussismilia* coral symbiont copes with heat stress. Bleaching events lead to the loss of symbiont pigments in the corals (Douglas, 2003). Heat stress induces changes in the cellular metabolic profile, oxidative state, cell structure, alterations in the central metabolism, signaling, and biosynthesis. Lipogenesis/lysis and membrane structure (Hillyer et al., 2016). However, it is not well understood the biochemical and cellular features of South Atlantic Ocean symbionts exposed to heat stress. Previous studies have demonstrated that thermal bleaching leads to damage to photosynthetic machinery (photosystem II) of the zooxanthellae, resulting in excess production of reactive oxygen species (ROS). In addition, the coral host (and zooxanthellae/endosymbiont) antioxidant defense strategies have been associated with the host-cell necrosis and detachment that underpins zooxanthellae expulsion (Gates et al., 1992; Dunn et al., 2002). Temperature elevation alone can damage Symbiodiniaceae cells in the host (Sammarco and Strychar, 2013). Nutrient uptake in symbionts differed under different temperatures due to stress susceptibility among corals hosting different symbionts (Baker et al., 2013).

The present study aimed to evaluate the effects of heat stress on the biochemical and cellular machinery of *Cladocopium* (subclade C3) during a 63-days. The integrated analyses of cell growth patterns, basic biochemical features (e.g., oxygen and protein production), lipidomic profiles, and transmission electron microscopy allowed us to gain new insights into the cell biology of *Cladocopium*.

Symbiodiniaceae cultures represent an important tool for studying this organism in the laboratory, including aspects of its physiology and cellular biology. This type of approach may help better understand the complex life cycle of Symbiodiniaceae. This organism has both a free-living and an endosymbiotic phase. Here we studied the free living phage under heat-stress conditions in controlled laboratory conditions.

## Materials and methods

### Cell culture, treatments, and density determination

*Cladocopium* C3 CCMR093 retrieved from the endemic coral *M. braziliensis* in Sebastião Gomes Reef, Abrolhos Bank, Brazil (Silva-Lima et al., 2015). CCMR093 cells were cultured in F/2 media (Guillard, 1975) at a density of 10^6^ cells mL^−1^ in a culture chamber at 24°C ± 1°C with a photon flux of *ca.* 80 μE/m2 /s, maintained under a photoperiod of 14-h light/10-h dark cycle over a period of 10 days. After this time, the experiment was performed with two treatments (i. control maintained at 24°C and ii. heat stress at 28°C) for 63 days. Each experiment was performed with 3 replicates. The experiment was repeated twice. A group of cells was placed in a culture chamber at 28°C ± 1°C in the same conditions described previously. During approximately 2 months the cells were collected in 1, 3,5, 7, 14, 21, 28, 35, 42, 49, 56, and 63 days and stored at −80°C for future analysis. Samples were collected on different days and fixed with acid Lugol’s solution (1% final concentration). *Cladocopium* cells were counted in most of the time every 7 days using a hemacytometer chamber and an inverted microscope at 200× magnification. Hemacytometer was loaded with 10 μl aliquots of *Cladocopium* cultures, and the four corner squares (each 1 mm2) of each 3 × 3-mm chamber were counted, for a total of three-chamber counts for each treatment were averaged.

### Pigment quantification and oxygen determinations

*Cladocopium* (C3) cells were collected in 1, 3,5, 7, 14, 21, 28, 35, 42, 49, 56 and 63 days and the cells were centrifuged at 3,000× *g* for 3 min. The supernatant was discarded and the pellet was subjected to chl a and c quantification. Chl was extracted in 90% acetone for 20 h in the dark at 4°C. The spectrophotometric analyses were performed in 96-well round-bottom plates (Corning Life Science) by Emax Plus microplate reader (Molecular Devices) and quantified using the equations of Jeffery and Humphrey (1975). For oxygen determinations, cells collected in the same conditions described previously were centrifuged at 3,000× *g* for 10 min. The supernatant was separated from the pellet and the supernatant was subjected to oxygen analysis. Dissolved oxygen was measured by Digimed DM 4P (Digicrom Analytical instrumentation-Ltda, Brazil).

### Protein, lipids contents, and extraction analysis

*Cladocopium* C3 cells were collected in 1, 3,5, 7, 14, 21, 28, 35, 42, 49, 56 and 63 days after temperature exposure in a centrifuge tube. The samples were centrifuged at 3000 g for 10 min. The supernatant was discarded and the pellet was resuspended with PBS 1X macerated with homogenizers for the 30s for the protein and lipids analyses. The protein content was analyzed by the method of Lowry et al. (1951) using bovine serum albumin (BSA) as standard and lipids were extracted using the method described by Bligh and Dyer (1959) and weighed by analytical balance (AUW 120D; Shimadzu, Kyoto, Japan).

The lipid extraction from samples was done according to Bligh and Dyer method (Bligh and Dyer, 1959). The lipid extracts were analyzed by one-dimensional High-Performance Thin-Layer Chromatography (HPTLC) on Silica Gel 60 plates (E. Merck, Darmstadt, Germany) for phospholipids has used a mixture of solvents consisting of acetone: methanol: acetic acid: chloroform: water (15:13:12:40:8 v/v; Horwitz and Perlman, 1987) for neutral lipids used n-hexane: diethyl ether: acetic acid (60,40:1 v/v; Vogel et al., 1962). Lipids were identified by comparison to commercial standards (Sigma-Aldrich Co.) and the relative lipid composition was determined by densitometry using Image Master TotalLab 1.11 (Amersham Pharmacia Biotech, England).

### Gas chromatography–mass spectrometry (GC/MS) analysis

The sterol fraction analysis was carried out as described by (Christie, 1989). Total lipids were dried with N2 and sterols extracted by saponification. Dried sterols from saponification were resuspended in 50 μl silylant BSTFA: TMCS 99:1 (Sigma-Aldrich) plus 50 μl pyridine and incubated for 1 h at 65°C. For the FA fraction lipid sample was dissolved in toluene (1 ml) in a tube, and 1% sulfuric acid in methanol (2 ml) was added. The mixture was left overnight in a stoppered tube at 50°C, then 1 ml of 5% containing sodium chloride was added and the required esters were extracted twice with 2 ml hexane using Pasteur pipettes to separate the layers. The solvent was removed in a stream of nitrogen. Dried FAME was resuspended in 50 μl hexane. GC/MS analysis was carried out on a Shimadzu GCMS-QP2010 Plus system, using an HP Ultra 2 (5% Phenyl - methylpolysiloxane), Agilent (25 m x 0.20 mm x 0.33 μm). Electron ionization (EI-70 eV) and a quadrupole mass analyzer, operated in scans from 40 to 440 amu to fatty acid fraction and 40 to 600 amu to sterols fractions. The components were identified by comparing their mass spectra with those of the library NIST05. Retention indices were also used to confirm the identity of the peaks in the chromatogram by Supelco 37 Component FAME Mix (Sigma-Aldrich). In these analyzes, we considered only molecules with an abundance greater than 1%.

### The transmission and scanning electron microscopy

*Cladocopium* (C3) cells were collected in 3, 14, and 35 days. To transmission electron microscopy (TEM) analysis, cells were fixed 2.5% glutaraldehyde in 0.1 M cacodylate buffer and 1.75% NaCl, pH 7.2 for 24 h. Scanning electron microscopy (SEM), cells adhered to Poly-L -lysine-coated (mol wt 300,000) glass coverslips and fixed for 1 h in the same solution previously described. After fixation, cells were washed in 0.1 M cacodylate buffer and postfixed for 1 h in 1% OsO4 containing 0.8% potassium ferrocyanide in 0.1 M cacodylate buffer (pH 7.2). Then for TEM analysis, the samples were washed in 0.1 M cacodylate buffer, dehydrated in acetone, and embedded in Epon. Ultrathin sections were stained with uranyl acetate and lead citrate and observed using Hitachi HT 7800 and Fei Tecnai Spirit transmission electron microscope. To SEM after postfixed with OsO4, cells were dehydrated in ethanol and critical point dried with liquid CO2. Finally, cells were coated with a 5 nm-thick layer of platinum and observed using a Zeiss Auriga 40 scanning electron microscope.

### Statistical analysis

Statistical analyses were performed using the Prism 8.0 program (GraphPad Software, San Diego, United States). Means were compared by two-way ANOVA. The values were considered significantly different when *p* < 0.05.

Non-metric multidimensional scaling (nMDS) analyses were used to display the cell abundance, and metabolic and lipidomic profiles from each time/temperature based on Bray–Curtis dissimilarity matrices (Walter et al., 2021). The nMDS result was plotted with the ggplot2 (Wickham, 2009) and reshaped (Wickham, 2007) packages.

To test the hypotheses that the response to abiotic data are different among temperature and days Permutational Multivariate Analysis of Variance (PERMANOVA) was performed using the “adonis” function of the Vegan package (Oksanen et al., 2005) using Bray-Curtis distances and 999 permutations.

## Results and discussion

### Heat stress reduces cell growth, pigment, and protein content In isolated *Cladocopium* CCMR093

**Figure 1:**
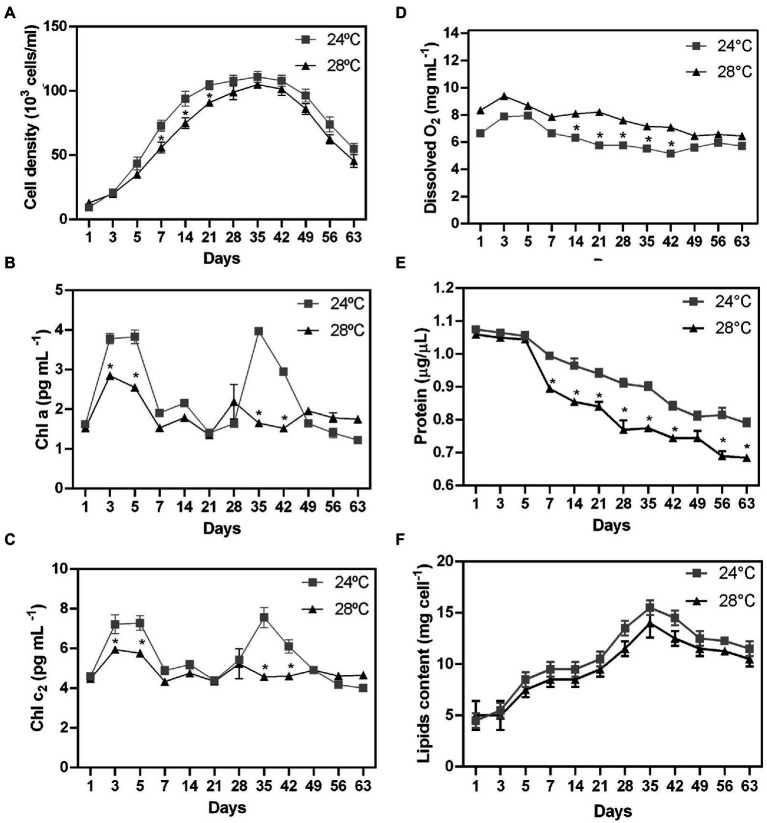
Biochemical changes during stress heated on different days in *Cladocopium* cells. To verify the effect of temperature on cellular biochemistry, CCMR093 cells cultured at 24°C and 28°C were submitted to analysis of growth curve **(A)**, pigments -Chla and chlc_2_ **(B,C)**, oxygen **(D)**, biomolecules-protein **(E)** and lipids **(F)**. Data are presented as the mean ± standard errors of the results of two independent experiments. Some error bars are obscured by data point markers. The statistical difference (*t*-Test) between bleaching (28°C) and control (24°C) is indicated as *p < 0.05.

There was a significant difference in cell growth rate between control and heat stress treatments after five days (Figure 1A; *p* < 0.01). Cell growth was observed until day 35 in both treatments (control: 96×10^3^ cells/mL; heat stressed: 74×10^3^ cells/mL). However, cell density decreased more rapidly in response to thermal stress (28°C) in comparison with the control treatment (24°C; Figure 1A; *p* < 0.01). The stationary phase was followed by a decrease in cell density (in both treatments) as a possible result of nutrient depletion in the culture medium (Li et al., 2011). Cell growth patterns may be also related to the clade type and differences in optimal growth temperature and photokinetics (Karim et al., 2015; Klueter et al., 2017). The growth rate decreased at high temperatures in thermally sensitive strains (Clade B and A), but not in thermally tolerant strains (Clade F and D; Karim et al., 2015). The reduction in growth rates may be a survival strategy for reallocating energy from cell growth to other metabolic functions such as the repair of damaged photosynthetic machinery or protection pathways.

To examine the possible effect of higher temperature on the CCMR093 cell pigment profile, cells were analyzed across the entire experimental period. There was a significant difference in pigment profile between control and heat stress treatments after 3 days. Chla and Chlc2 were reduced at 28°C (Figures 1B,C, respectively). The heat-stressed cells had lower levels of Chla (2.6 pg./cell) and Chlc2 (5.8 pg./cell), meanwhile, cells in the control treatment had 3.8 pg./cell Chla and 7.2 pg./cell Chlc2. On days 28 and 35, there was a second pigment peak with contents of Chla (1.6 pg./cell) and Chlc2 (4.5 pg./cell) lower in heat-stressed cells than the content of Chla (3.9 pg./cell) and Chlc2 (7.5 pg./cell) in the control group (Figures 1B,C), which may indicate a dynamic balance between photodamage and pigment repair. Heat stress-associated photobleaching may cause severe photoinhibition of PSII in Symbiodiniaceae (Takahashi et al., 2008).

Dissolved O_2_ values were significantly higher in the heat stress treatment after 1 day of the experiment (Figure 1D). Dissolved O_2_ values peaked on day 3 (control: 7.9 mg/l; heat stress: 8.3 mg/l; Figure 1D). On the other hand, the total protein cell levels were reduced in the heat stress treatment after 7 days (Figure 1E; *p* < 0.01). CCMR093 cells maintained at 24°C had a protein content of 0.79 to 1.0 μg/μL, while the cells maintained at 28°C had 0.68 to 0.89 μg/μL (Figure 1E). The results of this study suggest elevated temperature may increase oxygen concentration, and photosynthesis rates as observed previously in Symbiodiniaceae under conditions of heat stress (Fujise et al., 2014). However, the increased levels of reactive forms of oxygen may lead to deterioration of Symbiodiniaceae photosystems which may overwhelm antioxidant defenses to produce damage in the carbon fixation process and PS II (Lesser, 2006). The oxidative stress may also affect the protein content and result in lower protein levels in the cell observed here (Lesser, 2006). Meanwhile, the decrease in total cell protein levels may be a consequence of oxidative damage generated by the increase in cellular oxygen. Excess of reactive oxygen species (ROS), such as superoxide (O_2_^−^) and singlet oxygen (O_2_), may denature proteins, and cause damage to nucleic acids, lipids, membranes, and organelles (Lesser, 2006). The decrease in pigment and protein levels in the CCMR093 cells may be also related to increased levels of dissolved oxygen produced by CCMR093 cells under increased temperature. To further evaluate the effects of heat stress on CCMR093 cell physiology, a lipidomic analysis was performed.

### Identification of major lipidomic shifts driven by temperature stress

**Figure 2:**
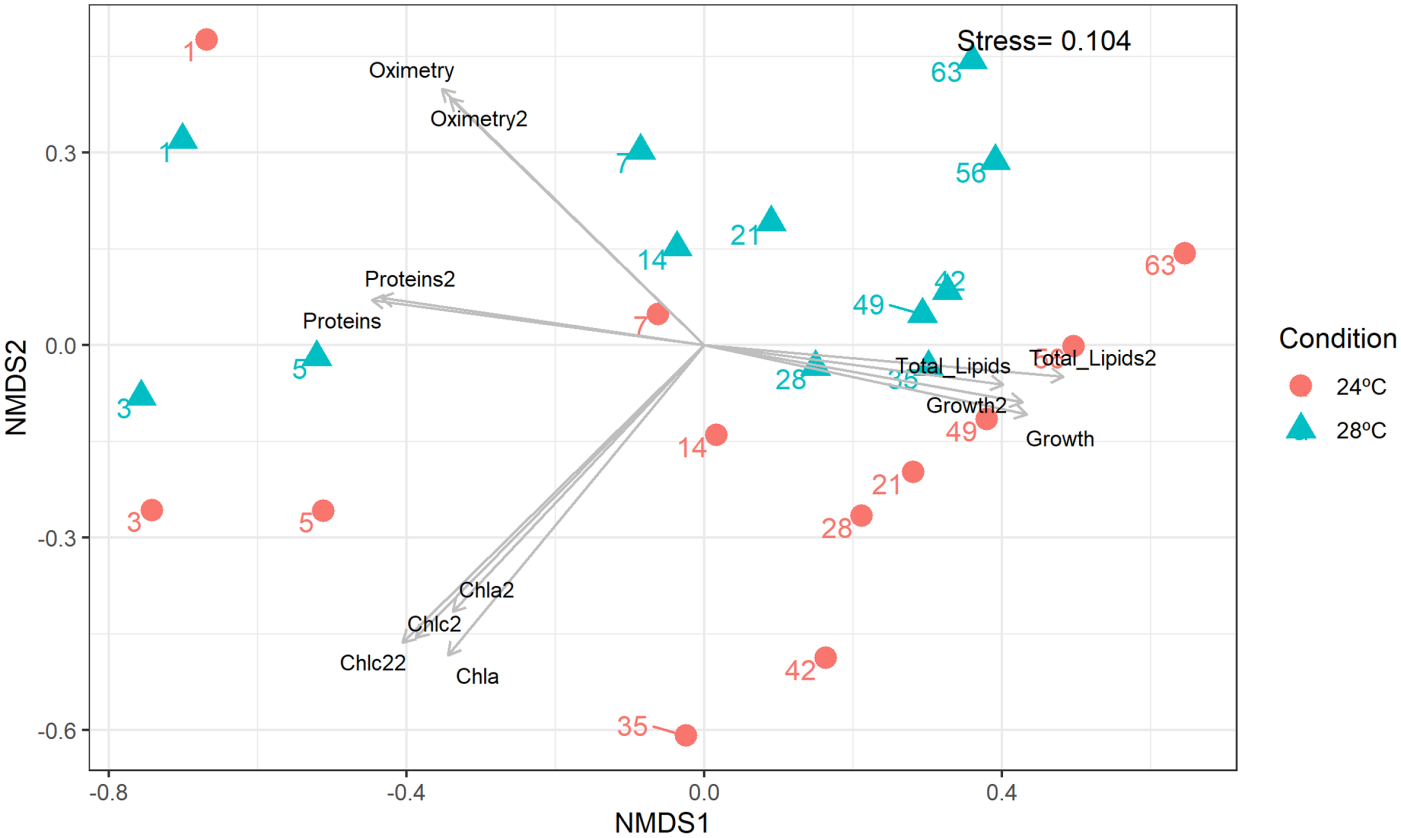
Non-metric multidimensional scaling (nMDS). Integrated cluster analysis using the cell abundance, metabolic and lipidomic profiles. nMDS plot representing the Bray–Curtis similarity of sample profiles from temperatures and days. Each dot represents a sample, the color represents the sampling temperature, and the arrows represent the nMDS abiotic characteristics scores.

The number of lipids gradually increased from day 3 (control: 8.5 mg; heat stress: 7.5 mg) reaching a peak on day 35 (control: 14 mg; heat stress: 12 mg; Figure 1F). The decrease in total lipids in the group subjected to heat stress was moderate compared with the control (Figure 1F). Lower lipid levels were observed in the heat stress over time which is consistent with a possible negative effect of higher temperature over cell lipid concentration and reduced amount of nutrients to the coral host (Hillyer et al., 2016). The Non-Metric Multidimensional Scaling (NDMS) analysis showed samples subjected to heat stress formed clusters when compared to all metabolic functions (Figure 2). When analyzing growth parameters, pigments, biomolecules, and oxygen, we can observe that samples subjected to thermal stress tend to be clustered (stress = 0.104). Further, PERMANOVA showed that the differences are accentuated over the days (df = 11, *F* = 87.353, R^2^ = 0.81166, *p* = 0.001; Supplementary Table S1).

**Figure 3:**
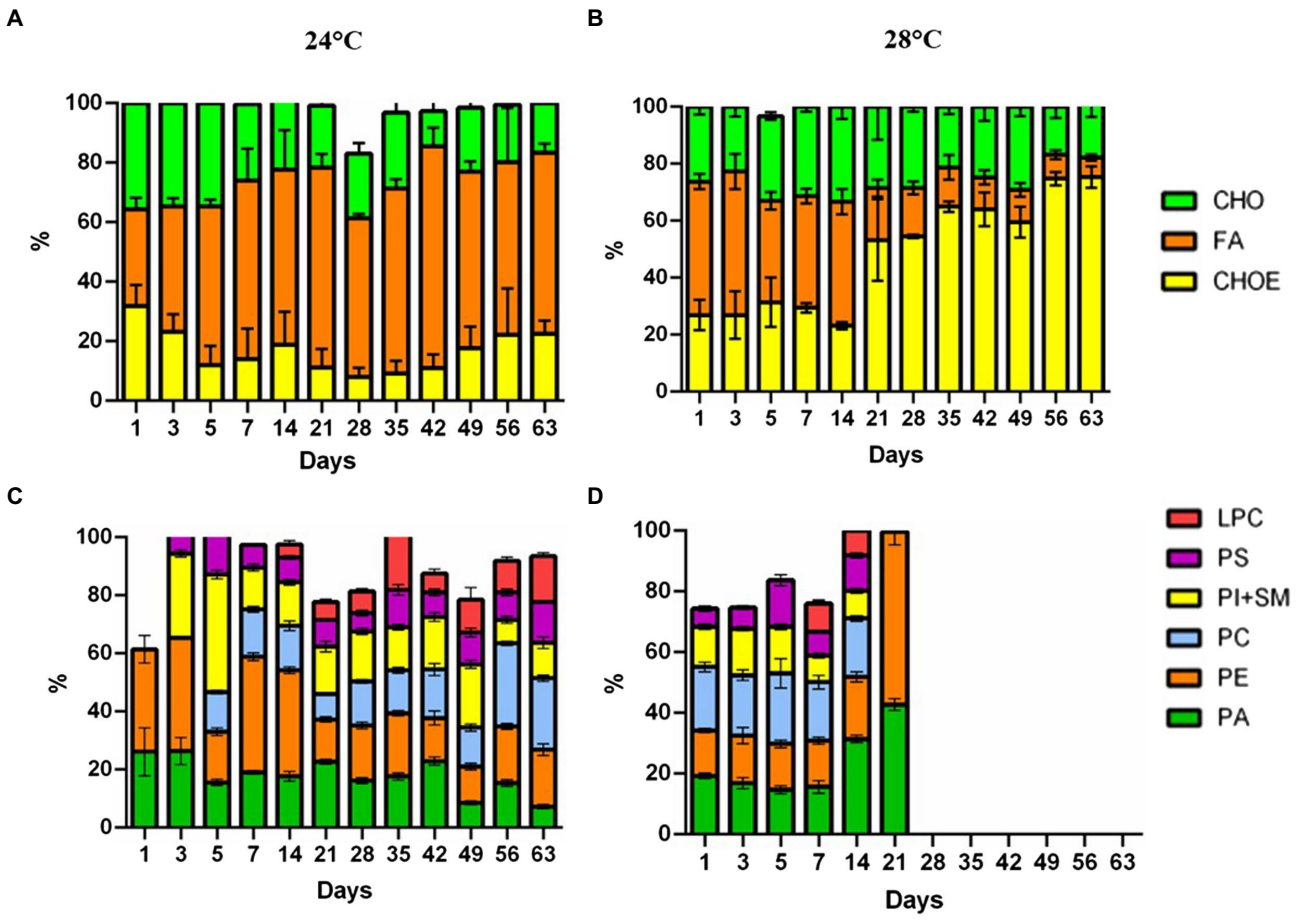
Lipidome analysis of *Cladocopium* cultured at 24°C and 28°C. Lipid composition in CCMR093 cells is expressed as the integral band values of HPTLC analysis. Panels **(A,B)** represent phospholipids and Panels **(C,D)** neutral lipids. LPC, lysophosphatidylcholine; SM, sphingomyelin; PI, phosphatidylinositol; PC, phosphatidylcholine; PA, phosphatidic acid; PS, phosphatidylserine and PE, phosphatidylethanolamine. MG, Monoacylglycerol; CHO, Cholesterol; FA, Free Fatty Acids; TG, Triacylglycerol; CHOE, Cholesteryl-esters; Unidentified lipids were not represented in the graph.

Cells in the control treatment (24°C) at the beginning of the assay had 32.5% fatty acid (FA). Over the days, there was an increase in FA in the cells, reaching values of 60.7%. Meanwhile, the heat stress treatment (28°C) had a decrease in fatty acids (46.8 to 6.8%; Figures 3A,B, respectively). Neutral lipid classes, including cholesterol (CHO), and cholesteryl-esters (CHOE) increased in the heat-stress treatment (26.9 to 75.4%; Figure 3B). Cells in the heat-stress treatment had an increase in phosphatidic acid (PA) (19.2% day 1 to 42.7% day 21), and phosphatidylethanolamine (PE) (14.9% day 1 to 56.8% day 21; Figure 3D). These cells had a reduction in phosphatidylinositol and sphingomyelin (SM + PI; 13.3% day 1 to 9.1% day 14; Figure 3D). Phospholipid classes were absent from day 28 onwards. On the contrary, cells in the control treatment had major phospholipids.

Changes in the lipidomic profile of cells under heat stress, such as the decrease in free fatty acids and increase in esterified cholesterol may be a result of the accumulation of lipid droplets within the CCMR093. Cells under stress may accumulate large amounts of some lipid classes, such as triacylglycerol, sterols, cholesterol ester, phosphatidylcholine, fatty acid, phosphatidylinositol, and phosphatidylethanolamine (Peng et al., 2011). Lipids are important for the formation of structural components but appear not essential for nutrition and energy storage in Symbiodioniaceae (Gordon and Leggat, 2010). Healthy coral cells and Symbiodiniaceae cells have a proportion of neutral lipids/phospholipids of 75%/16 and 8%/67%, respectively (Patton et al., 1977). However, under heat stress conditions, these proportions are altered due to a decrease in the translocation of molecules between the host and the symbiont which may affect host nutrition (Wooldridge, 2013). Furthermore, remodeling of membrane lipids and other cell components may represent cellular responses to cope with heat stress (Tchernov et al., 2004). To further elucidate the types of lipids and possible heat stress biomarkers, GC–MS analysis was performed.

**Figure 4:**
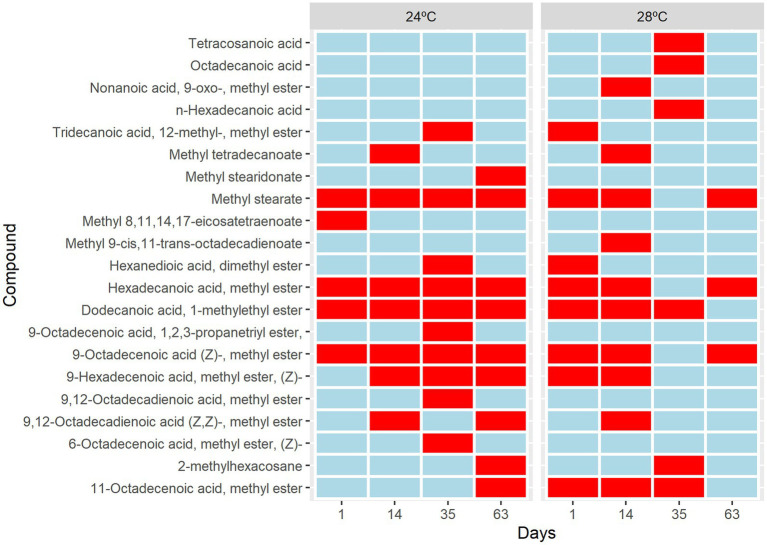
Metabolic changes in *Cladocopium* cultured at 24°C and 28°C. Heat map analysis showing the intensity of fatty acids composition in *Cladocopium* during 1, 14, 35, and 63 days. The Colour bar indicates levels of metabolite, the red colour indicates upregulation, and the blue colour indicates downregulation.

Five different sterol species and 21 different fatty acids (FA) species 12 saturated (SFA), 5 monounsaturated (MUFA), and 4 polyunsaturated (PUFA) were identified (Supplementary Table S2). The short-term metabolic changes of fatty acids in *Cladocopium* C3 were observed from the first day that the cells were subjected to heat stress. There was an increase in saturation and a decrease in fatty acid unsaturation over time under heat stress (Figure 4). An upregulation of molecule 11octadecenoic acid-methyl ester was observed (Figure 4). However, the greatest changes occurred from day 35, where the metabolic profile of cells under stress conditions is quite different from the control group. In these cells there is suppression of many fatty acids and the appearance of unique molecules such as tetracosanoic acid, octadecanoic acid, and n-hexadecanoic acid (Figure 4). On day 63, fatty acid suppression is even greater with the appearance of only 3 molecules (Figure 4). Cells subjected to heat stress showed suppression of sterols (Supplementary Figure S1). Our results showed that the composition of fatty acids and sterols varied in cells subjected to heat stress. Over time, many PUFAs compounds remained in smaller amounts or disappeared during the heat stress treatment. Fatty acids are key structural components of cell membranes involved in thermal and photo-acclimation processes. Previous studies demonstrated that high temperatures could decrease the unsaturation levels of photosynthetic membrane lipids and that this would have an effect on the thermal stability of PSII and could be involved in a thermal tolerance mechanism (Sato et al., 1996). In addition, the high saturation rate found in fatty acids may be a strategy to prevent leakage of biological membranes that occur at high temperatures (Gombos et al., 1994). A decrease in fatty acids, cholesterol, and 4-methyl sterol was observed (Kneeland et al. (2013). The presence of mono- and digalactosyldiacylglycerols (MGDG and DGDG), and lower unsaturation of sulfoquinovosyldiacylglycerol (SQDG) were also observed in coral endosymbionts (Sikorskaya et al., 2021). The lipidomic analyses indicated that cellular changes may have occurred in the CCMR093. To further investigate possible cellular responses to heat stress, a detailed electronic microscopy analysis was performed.

### Increased temperature induces cellular changes in *Cladocopium* C3

**Figure 5:**
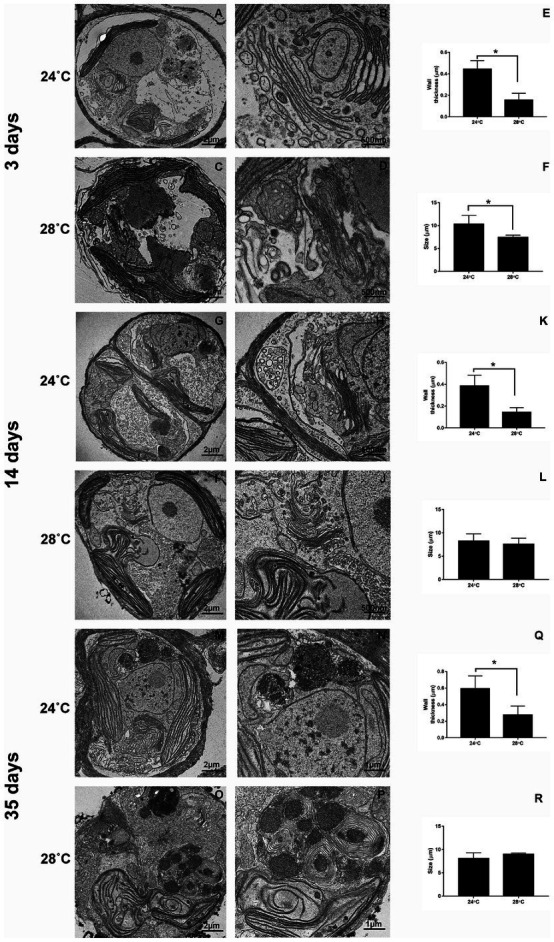
Ultrastructural changes during heat stress in *Cladocopium* cells. TEM micrographs showing *Cladocopium* cells cultured at 3 days were demonstrated in panels **A** and **B** (24°C) and **C** and **D** (28°C), 14 days- Panels **G** and **H** (24°C) and **I** and **J** (28°C), and 35 days- Panels **M** and **N** (24°C) and **O** and **P** (28°C). *Cladocopium* Wall thickness (**E**, **K**, and **Q**) and Size (**F**, **L**, and **R**) were represented in graphs. Abbreviations: LD, or * lipid droplet; Ch, chloroplast; Pyr, pyrenoids; N, nucleolus; W, wall; AC, Accumulation body; GC, Golgi Complex. The scale bars indicate in the images.

CCMR093 cells demonstrated a well-defined cellular organization with a normal cell wall and the presence of chloroplasts, nucleus, Golgi complex, and pyrenoid after 3 days of treatment (24°C; Figures 5A,B,E,F). However, cellular changes were observed in cells exposed to heat stress (28°C) on day 3, which included elongated and subtly misshapen chloroplasts, many mitochondria, and thinner cell walls (Figures 5C–F). There was a clear increase in the size of the nucleus and change in the morphology of the Golgi complex, chloroplasts, and an increase in the number of lipid droplets after 14 days experiment. Cells demonstrated clear signs of damage with the presence of fewer organelles and cytoplasmic debris in the lumen, a decrease in nucleus and pyrenoid size, distortion of the thylakoid membrane, and a considerable increase in lipid droplets after 35 days of the experiment (Figure 5). The accumulation of lipid bodies over time was a possible effect of heat stress. Lipid droplet increase in Symbiodiniaceae was associated with coral bleaching and reduced exportation to the host by the Symbiodiniaceae (Peng et al., 2011). In addition, under partial or severe bleaching conditions, a gradual increase in the size of lipid droplets and changes in the proportion of lipid content can be observed (Luo et al., 2009). The scanning microscopy showed differences after 3 days of heat exposure. The cell surface of organisms subjected to heat stress showed a smoother appearance when compared to the control. Over time, there was an appearance of irregularities with a wilted appearance on the cell surface and many filamentous aggregates indicating cell damage (Supplementary Figure S2).

The present study demonstrated that thermal stress negatively affects isolated *Cladocopium* spp. from *Mussismilia* coral.

Recent studies, support the idea that algal symbionts are less tolerant to heat stress than their coral hosts and that damage to algal photosynthetic apparatus leads to bleaching in corals (Fitt et al., 2001; Bhagooli and Hidaka, 2003). The estimated order of bleaching susceptibility was also different between host and isolated zooxanthellae (Bhagooli and Hidaka, 2003). This also suggests that host tissue affects the stress susceptibility of zooxanthellae through photoprotection mechanisms. Zooxanthellae that are highly protected from photodamage by coral hosts may display high susceptibility to stresses when isolated from hosts (Bhagooli and Hidaka, 2003).

Damage to the photosynthetic apparatus observed here may lead to *Cladocopium* death which reinforces the negative effect of thermal stress on free-living *Cladocopium*. Bleaching and other negative effects of temperature stress in the host have been shown earlier which reinforces the findings of the present study (Hughes et al., 2017; Lisboa et al., 2018).

## Concluding remarks

The effects of elevated temperature on *Cladocopium* cells are observed a few days after heat-stress which indicates that short-term thermal anomalies are detrimental to *Mussismilia* coral holobiont health. The results of the present study clearly demonstrate the increase in lipid contents over time and the intracellular accumulation of these molecules in lipid droplets in the CCRM093. The observed biochemical and cellular modifications may affect the host’s nutrition, hampering the flow of nutrients from the *Cladocopium* symbiont to the *Mussismilia* coral host. Possibly, heat stress is detrimental to the health of this South Atlantic Ocean coral.

## Data availability statement

The original contributions presented in the study are included in the article/Supplementary material, further inquiries can be directed to the corresponding authors.

## Author contributions

ML: conceptualization, formal analysis, investigation, methodology, original draft, writing – review and editing. LH: formal analysis, visualization, original draft and review. TS and DT: formal analysis. MC: formal analysis, investigation and methodology. TV: writing and review. GA: conceptualization and supervision. WS: methodology, validation, visualization and review. FT and CT: project administration, resources, validation, visualization, writing – original draft, writing – review and editing. All authors contributed to the article and approved the submitted version.

## Conflict of interest

The authors declare that the research was conducted in the absence of any commercial or financial relationships that could be construed as a potential conflict of interest.

## Publisher’s note

All claims expressed in this article are solely those of the authors and do not necessarily represent those of their affiliated organizations, or those of the publisher, the editors and the reviewers. Any product that may be evaluated in this article, or claim that may be made by its manufacturer, is not guaranteed or endorsed by the publisher.

## References

[ref1] Antonelli P. Rutz S. Sammarco P. Strychar K. (2015). Evolution of synbiosis in hermatypic corals: a model of the past, present and future. Nonl. Anal. 32, 389–402. doi: 10.13140/RG.2.1.4549.2968

[ref2] Ayalon I. Benichou J. I. C. Avisar D. Levy O. (2021). The endosymbiotic coral algae Symbiodiniaceae are sensitive to a sensory pollutant: artificial light at night, ALAN. Front. Physiol. 12:695083. doi: 10.3389/fphys.2021.695083, PMID: 34234696 PMC8256845

[ref3] Baker A. (2003). Flexibility and specificity in coral-algal symbiosis: diversity, ecology, and biogeography of *Symbiodinium*. Annu. Rev. Ecol. Evol. Syst. 34, 661–689. doi: 10.1146/annurev.ecolsys.34.011802.132417

[ref4] Baker D. M. Andras J. P. Jordán-Garza A. G. Fogel M. L. (2013). Nitrate competition in a coral symbiosis varies with temperature among *Symbiodinium* clades. ISME J. 7, 1248–1251. doi: 10.1038/ismej.2013.12, PMID: 23407311 PMC3660672

[ref5] Baker A. Glynn P. Riegl B. (2008). Climate change and coral reef bleaching: an ecological assessment of long-term impacts, recovery trends and future outlook. Estuar. Coast. Shelf Sci. 80, 435–471. doi: 10.1016/j.ecss.2008.09.003

[ref6] Bhagooli R. Hidaka M. (2003). Comparison of stress susceptibility of in hospite and isolated zooxanthellae among five coral species. J. Exp. Mar. Biol. Ecol. 291, 181–197. doi: 10.1016/S0022-0981(03)00121-7

[ref7] Bligh E. G. Dyer W. J. (1959). A rapid method of total lipid extraction and purification. Can. J. Biochem. Physiol. 37, 911–917. doi: 10.1139/o59-099 13671378

[ref8] Christie W. W. (1989). *Gas chromatography and lipids*. PJ Barnes & Associates (The Oily Press Ltd); Chapter 4, in press.

[ref9] Douglas A. E. (2003). Coral bleaching--how and why? Mar. Pollut. Bull. 46, 385–392. doi: 10.1016/S0025-326X(03)00037-7 12705909

[ref10] Dunn S. R. Bythell J. C. Le Tissier M. D. A. Burnett W. J. Thomason J. C. (2002). Programmed cell death and cell necrosis activity during hyperthermic stress-induced bleaching of the symbiotic sea anemone *Aiptasia* sp. J. Exp. Mar. Biol. Ecol. 272, 29–53. doi: 10.1016/S0022-0981(02)00036-9

[ref11] Fitt W. K. Brown B. E. Warner M. E. Dunne R. P. (2001). Coral bleaching: interpretation of thermal tolerance limits and thermal thresholds in tropical corals. Coral Reefs 20, 51–65. doi: 10.1007/s003380100146

[ref12] Fujise L. Yamashita H. Suzuki G. Sasaki K. Liao L. M. Koike K. (2014). Moderate thermal stress causes active and immediate expulsion of photosynthetically damaged zooxanthellae (*Symbiodinium*) from corals. PLoS One 9:e114321. doi: 10.1371/journal.pone.0114321, PMID: 25493938 PMC4262390

[ref13] Gates R. D. Baghdasarian G. Muscatine L. (1992). Temperature stress causes host cell detachment in symbiotic cnidarians: implications for coral bleaching. Biol. Bull. 182, 324–332. doi: 10.2307/1542252, PMID: 29304594

[ref500] Gombos Z. Wada H. Hideg E. Murata N. (1994). The unsaturation of membrane lipids stabilizes photosynthesis against heat stress. Plant Physiol. 104, 563–567., PMID: 12232106 10.1104/pp.104.2.563 PMC159232

[ref14] Gordon B. R. Leggat W. (2010). *Symbiodinium*-invertebrate symbioses and the role of metabolomics. Mar. Drugs 8, 2546–2568. doi: 10.3390/md8102546, PMID: 21116405 PMC2992991

[ref15] Guillard R.R.L. Culture of phytoplankton for feeding marine invertebrates in “Culture of Marine Invertebrate Animals.” (1975). Smith W.L. Chanley M.H.) Plenum Press, New York, USA. pp. 26–60.

[ref16] Hillyer K. E. Tumanov S. Villas-Bôas S. Davy S. K. (2016). Metabolite profiling of symbiont and host during thermal stress and bleaching in a model cnidarian-dinoflagellate symbiosis. J. Exp. Biol. 219, 516–527. doi: 10.1242/jeb.128660, PMID: 26685173

[ref17] Hoegh-Guldberg O. Bruno J. F. (2010). The impact of climate change on the world's marine ecosystems. Science 328, 1523–1528. doi: 10.1126/science.1189930 20558709

[ref18] Hoegh-Guldberg O. Mumby P. J. Hooten A. J. Steneck R. S. Greenfield P. Gomez E.. (2007). Coral reefs under rapid climate change and ocean acidification. Science 318, 1737–1742. doi: 10.1126/science.1152509, PMID: 18079392

[ref19] Horwitz J. Perlman R. L. (1987). Measurement of inositol phospholipid metabolism in PC12 pheochromocytoma cells. Methods Enzymol. 141, 169–175. doi: 10.1016/0076-6879(87)41065-3, PMID: 3298960

[ref20] Hughes T. P. Kerry J. T. Álvarez-Noriega M. Álvarez-Romero J. G. Anderson K. D. Baird A. H.. (2017). Global warming and recurrent mass bleaching of corals. Nature 543, 373–377. doi: 10.1038/nature21707, PMID: 28300113

[ref21] Masson-Delmotte V. Zhai P. Pörtner H. O. Roberts D. Skea J. Shukla P. R. (eds). (2018). “Global warming of 1.5°C,” in An IPCC special report on the impacts of global warming of 1.5°C above pre-industrial levels and related global greenhouse gas emission pathways, in the context of strengthening the global response to the threat of climate change, sustainable development, and efforts to eradicate poverty. (Geneva: World Meteorological Organization).

[ref22] Janouškovec J. Gavelis G. S. Burki F. Dinh D. Bachvaroff T. R. Gornik S. G.. (2017). Major transitions in dinoflagellate evolution unveiled by phylotranscriptomics. Proc. Natl. Acad. Sci. U. S. A. 114, E171–E180. doi: 10.1073/pnas.1614842114, PMID: 28028238 PMC5240707

[ref23] Jeffery S. W. Humphrey G. F. (1975). New spectrophotometric equations for determining chlorophylls a, b, c1, and c2 in higher plants, algae and natural phytoplankton. Biochem. Physiol. Pflanz. 167, 191–194. doi: 10.1016/S0015-3796(17)30778-3

[ref24] Jiang P. L. Pasaribu B. Chen C. S. (2014). Nitrogen-deprivation elevates lipid levels in *Symbiodinium* spp. by lipid droplet accumulation: morphological and compositional analyses. PLoS One 9:e87416. doi: 10.1371/journal.pone.0087416, PMID: 24475285 PMC3903884

[ref25] Jokiel P. Brown E. (2004). Global warming, regional trends and inshore environmental conditions influence coral bleaching in Hawaii. Glob. Chang. Biol. 10, 1627–1641. doi: 10.1111/j.1365-2486.2004.00836.x

[ref26] Karim W. Nakaema S. Hidaka M. (2015). Temperature effects on the growth rates and photosynthetic activities of *Symbiodinium* cells. J. Mar. Sci. Eng. 3, 368–381. doi: 10.3390/jmse3020368

[ref27] Klueter A. Trapani J. Archer F. I. McIlroy S. E. Coffroth M. A. (2017). Comparative growth rates of cultured marine dinoflagellates in the genus Symbiodinium and the effects of temperature and light. PLoS One 12:e0187707. doi: 10.1371/journal.pone.0187707, PMID: 29186143 PMC5706665

[ref28] Kneeland J. Hughen K. Cervino J. Salas H. Briana B. Eglinton T. (2013). Lipid biomarkers in *Symbiodinium* dinoflagellates: new indicators of thermal stress. Coral Reefs 32, 923–934. doi: 10.1007/s00338-013-1076-3

[ref29] LaJeunesse T. C. Parkinson J. E. Gabrielson P. W. Jeong H. J. Reimer J. D. Voolstra C. R.. (2018). Systematic revision of *Symbiodiniaceae* highlights the antiquity and diversity of coral endosymbionts. Curr. Biol. 28, 2570–2580.e6. doi: 10.1016/j.cub.2018.07.008, PMID: 30100341

[ref30] Lesser M. P. (2006). Oxidative stress in marine environments: biochemistry and physiological ecology. Annu. Rev. Physiol. 68, 253–278. doi: 10.1146/annurev.physiol.68.040104.110001, PMID: 16460273

[ref31] Li X. Hu H. Y. Zhang Y. P. (2011). Growth and lipid accumulation properties of a freshwater microalga *Scenedesmus* sp. under different cultivation temperature. Bioresour. Technol. 102, 3098–3102. doi: 10.1016/j.biortech.2010.10.055, PMID: 21055924

[ref32] Lisboa D. Kikuchi R. Leao Z. (2018). El Niño, sea surface temperature anomaly and coral bleaching in the South Atlantic: a chain of events modeled with a Bayesian approach. J. Geophys. Res. Oceans 123, 2554–2569. doi: 10.1002/2017JC012824

[ref33] Lowry O. H. Rosebrough N. J. Farr A. L. Randall R. J. (1951). Protein measurement with the Folin phenol reagent. J. Biol. Chem. 193, 265–275. doi: 10.1016/S0021-9258(19)52451-6, PMID: 14907713

[ref34] Luo Y.-J. Wang L.-H. Chen W. Peng S.-E. Tzen J. Hsiao Y.. (2009). Ratiometric imaging of gastrodermal lipid bodies in coral–dinoflagellate endosymbiosis. Coral Reefs 28, 289–301. doi: 10.1007/s00338-008-0462-8

[ref35] Mies M. Francini-Filho R. Zilberberg C. Garrido A. Longo G. Laurentino E.. (2020). South atlantic coral reefs are major global warming refugia and less susceptible to bleaching. Front. Mar. Sci. 7:514. doi: 10.3389/fmars.2020.00514

[ref36] Muller-Parker G. Davy S. (2005). Temperate and tropical algal-sea anemone symbioses. Invertebr. Biol. 120, 104–123. doi: 10.1111/j.1744-7410.2001.tb00115.x

[ref37] Nitschke M. Davy S. Cribb T. Ward S. (2014). The effect of elevated temperature and substrate on free-living *Symbiodinium* cultures. Coral Reefs 34, 161–171. doi: 10.1007/s00338-014-1220-8

[ref38] Oksanen J Kindt R O’Hara B. (2005). Vegan: R functions for vegetation ecologists. Available at: http://cc.oulu.fi/jarioksa/softhelp/vegan.html

[ref39] Pasaribu B. Li Y.-S. Kuo P.-C. Lin I. P. Tew K. Tzen J.. (2016). The effect of temperature and nitrogen deprivation on cell morphology and physiology of Symbiodinium. Oceanologia 58, 272–278. doi: 10.1016/j.oceano.2016.04.006

[ref40] Patton J. S. Abraham S. Benson A. A. (1977). Lipogenesis in the intact coral *Pocillopora capitata* and its isolated zooxanthellae: evidence for a light-driven carbon cycle between symbiont and host. Mar. Biol. 44, 235–247. doi: 10.1007/BF00387705

[ref41] Peng S. E. Chen W. N. Chen H. K. Lu C. Y. Mayfield A. B. Fang L. S.. (2011). Lipid bodies in coral-dinoflagellate endosymbiosis: proteomic and ultrastructural studies. Proteomics 11, 3540–3555. doi: 10.1002/pmic.201000552, PMID: 21751349

[ref42] Picciani N. de Lossio G. ESeiblitz I. Paiva P. Castro C. Zilberberg C. (2016). Geographic patterns of *Symbiodinium* diversity associated with the coral *Mussismilia hispida* (Cnidaria, Scleractinia) correlate with major reef regions in the Southwestern Atlantic Ocean. Mar. Biol. 163:236. doi: 10.1007/s00227-016-3010-z

[ref43] Reichert J. Tirpitz V. Anand R. Bach K. Knopp J. Schubert P.. (2021). Interactive effects of microplastic pollution and heat stress on reef-building corals. Environ. Pollut. 290:118010. doi: 10.1016/j.envpol.2021.118010, PMID: 34488160

[ref44] Reis V. M. Karez C. S. Mariath R. de Moraes F. C. de Carvalho R. T. Brasileiro P. S.. (2016). Carbonate production by benthic communities on shallow Coralgal reefs of Abrolhos Bank, Brazil. PLoS One 11:e0154417. doi: 10.1371/journal.pone.0154417, PMID: 27119151 PMC4847907

[ref45] Ruiz-Moreno D. Willis B. L. Page A. C. Weil E. Cróquer A. Vargas-Angel B.. (2012). Global coral disease prevalence associated with sea temperature anomalies and local factors. Dis. Aquat. Org. 100, 249–261. doi: 10.3354/dao02488, PMID: 22968792

[ref46] Sammarco P. W. Strychar K. B. (2013). Responses to high seawater temperatures in zooxanthellate octocorals. PLoS One 8:e54989. doi: 10.1371/journal.pone.0054989, PMID: 23405104 PMC3566138

[ref47] Sato N. Sonoike K. Kawaguchi A. Tsuzuki M. (1996). Contribution of lowered unsaturation levels of chloroplast lipids to high temperature tolerance of photosynthesis in *Chlamydomonas reinhardtii*. J. Photochem. Photobiol. B Biol. 36, 333–337. doi: 10.1016/s1011-1344(96)07389-7

[ref48] Sikorskaya T. V. Efimova K. V. Imbs A. B. (2021). Lipidomes of phylogenetically different symbiotic dinoflagellates of corals. Phytochemistry 181:112579. doi: 10.1016/j.phytochem.2020.112579, PMID: 33166751

[ref49] Silva-Lima A. W. Froes A. M. Garcia G. D. Tonon L. A. C. Swings J. Cosenza C. A. N.. (2021). Mussismilia braziliensis white plague disease is characterized by an affected coral immune system and dysbiosis. Microb. Ecol. 81, 795–806. doi: 10.1007/s00248-020-01588-5, PMID: 33000311

[ref50] Silva-Lima A. W. Walter J. M. Garcia G. D. Ramires N. Ank G. Meirelles P. M.. (2015). Multiple *Symbiodinium* strains are hosted by the Brazilian endemic corals *Mussismilia* spp. Microb. Ecol. 70, 301–310. doi: 10.1007/s00248-015-0573-z, PMID: 25666537

[ref51] Stanley G. D. Jr. (2006). Ecology. Photosymbiosis and the evolution of modern coral reefs. Science 312, 857–858. doi: 10.1126/science.1123701, PMID: 16690848

[ref52] Suggett D. J. Warner M. E. Leggat W. (2017). Symbiotic dinoflagellate functional diversity mediates coral survival under ecological crisis. Trends Ecol. Evol. 32, 735–745. doi: 10.1016/j.tree.2017.07.013, PMID: 28843439

[ref53] Takahashi S. Whitney S. Itoh S. Maruyama T. Badger M. (2008). Heat stress causes inhibition of the de novo synthesis of antenna proteins and photobleaching in cultured *Symbiodinium*. Proc. Natl. Acad. Sci. U. S. A. 105, 4203–4208. doi: 10.1073/pnas.0708554105, PMID: 18322010 PMC2393757

[ref54] Tchernov D. Gorbunov M. Y. de Vargas C. Narayan Yadav S. Milligan A. J. Häggblom M.. (2004). Membrane lipids of symbiotic algae are diagnostic of sensitivity to thermal bleaching in corals. Proc. Natl. Acad. Sci. U. S. A. 101, 13531–13535. doi: 10.1073/pnas.0402907101, PMID: 15340154 PMC518791

[ref55] Varasteh T. Salazar V. Tschoeke D. Francini-Filho R. Swings J. Garcia G.. (2021). *Breviolum* and *Cladocopium* are dominant among symbiodiniaceae of the coral holobiont *Madracis decactis*. Microb. Ecol. 84, 325–335. doi: 10.1007/s00248-021-01868-8, PMID: 34561754

[ref56] Varasteh T. Shokri M. Rajabi-Maham H. Behzadi S. Hume B. (2018). *Symbiodinium thermophilum* symbionts in *Porites harrisoni* and *Cyphastrea microphthalma* in the northern Persian Gulf, Iran. J. Mar. Biol. Associat. UK 98, 2067–2073. doi: 10.1017/S0025315417001746

[ref57] Vogel W. C. Zieve L. Carleton R. O. (1962). Measurement of serum lecithin, lysolecithin, and sphingomyelin by a simplified chromatographic technique. J. Lab. Clin. Med. 59, 335–344. 13926441

[ref58] Walter J. Oliveira L. Tschoeke D. Meirelles P. Neves M. Batista D.. (2021). Metagenomic insights into ecosystem function in the microbial Mats of a large hypersaline coastal lagoon system. Front. Mar. Sci. 8:715335. doi: 10.3389/fmars.2021.715335

[ref59] Weng L. C. Pasaribu B. Lin I. P. Tsai C. H. Chen C. S. Jiang P. L. (2014). Nitrogen deprivation induces lipid droplet accumulation and alters fatty acid metabolism in symbiotic dinoflagellates isolated from Aiptasia pulchella. Sci. Rep. 4:5777. doi: 10.1038/srep05777, PMID: 25047647 PMC4105741

[ref60] Wickham H. (2007). Reshaping data with the reshape package. J. Stat. Softw. 21, 1–20. doi: 10.18637/jss.v021.i12

[ref61] Wickham H. (2009). ggplot2-elegant graphics for data analysis. New York, NY: Springer.

[ref62] Wooldridge S. (2013). Breakdown of the coral-algae symbiosis: towards formalizing a linkage between warm-water bleaching thresholds and the growth rate of the intracellular zooxanthellae. Biogeosciences 10, 1647–1658. doi: 10.5194/bg-10-1647-2013

