# Supplementary material for: Insights on the biochemical and cellular changes induced by heat stress in the Cladocopium isolated from coral Mussismilia braziliensis
Source: Front Microbiol. 2022 Oct 10;13:973980. doi: 10.3389/fmicb.2022.973980 (PMC9590694; doi:10.3389/fmicb.2022.973980)
Supplement: Supplementary file 1 [file Data_Sheet_1.pdf]

Supplementary Material

Table S1: Permutational Multivariate Analysis of Variance

	Df	SumOfSqs	R^2	F	Pr(>F)
Treatment	1	0.17553	0.09543	112.971	0.001
Days	11	149.297	0.81166	87.353	0.001
Residual	11	0.17091	0.09292		
Total	23	183.941	100.000		

Table S2: Composition of fatty acid and sterols in *Cladocopium* C3

SUFAs	MUFAs	PUFAs	Sterols
Hexanedioic acid, dimethyl ester	9-Hexadecenoic acid, methyl ester, (Z)-	Methy l8,11,14,17-eicosatetraenoate	Stigmasterol
Methyl stearate	9-Octadecenoic acid (Z)-, methyl ester	9,12-Octadecadienoic acid (Z,Z)-, methyl ester	Ergosta-5,22-dien-3-ol, (3.beta.,22E)-
Methyl stearidonate	11-Octadecenoic acid, methyl ester	9,12-Octadecadienoic acid, methyl ester	Cholest-5-en-3-ol,24-propylidene-, (3.beta.)-
Methyl tetradecanoate	6-Octadecenoic acid, methyl ester, (Z)-	Methy l9-cis,11-trans-octadecadienoate	Cholesterol
Tridecanoic acid, 12-methyl-, methyl ester	9-Octadecenoic acid, 1,2,3-propanetriyl ester,		Stigmasta-5,24(28)-dien-3-ol, (3.beta.)-
Dodecanoic acid, 1-methylethyl ester			
2-methyloctacosane			
Octadecanoic acid			
Hexadecanoic acid, methyl ester			
Nonanoic acid, 9-oxo-, methyl ester			
Tetracosanoic acid			
Octadecanoic acid			

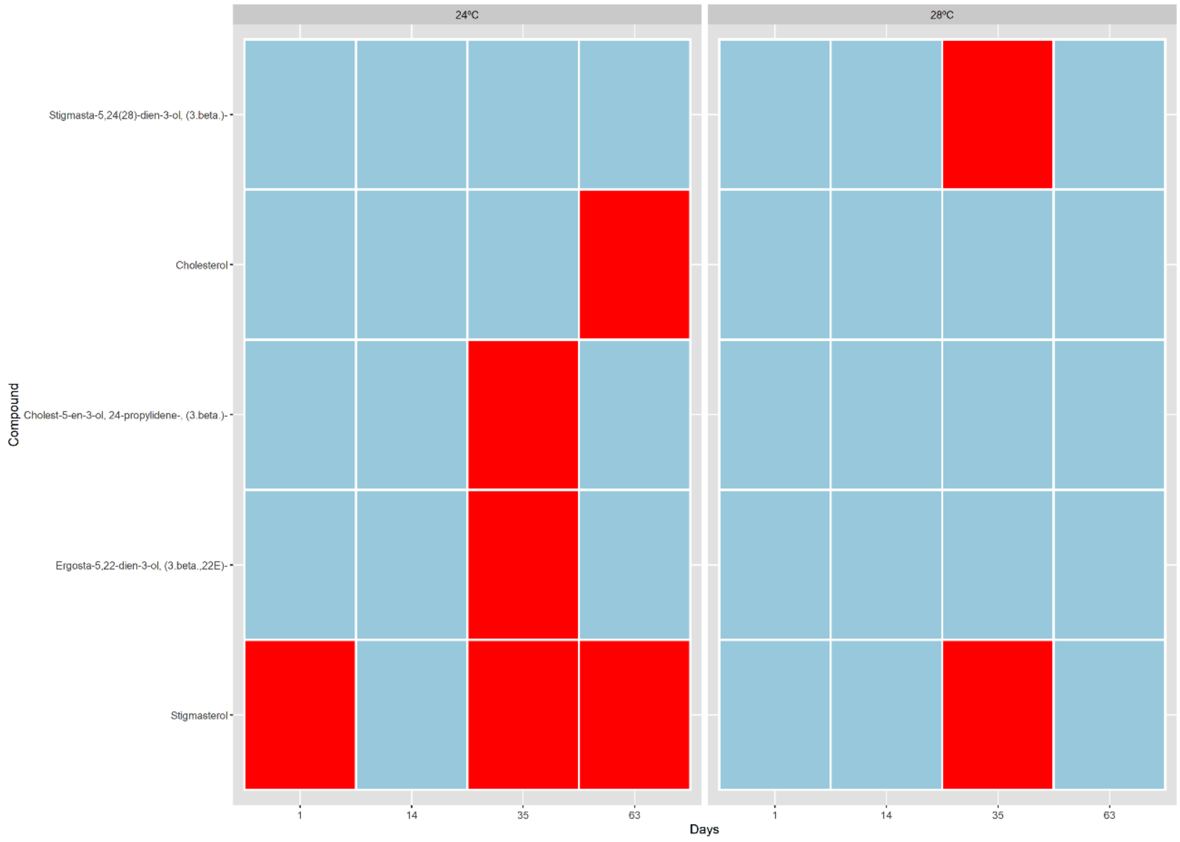

Fig.S1: Metabolic changes in *Cladocopium* cultured at 24°C and 28°C. Heat map analysis showing intensity of sterols composition in *Cladocopium* during 1, 14, 35 and 63 days. Colour bar indicates levels of metabolite, red colour indicates upregulation and blue colour indicates down regulation.

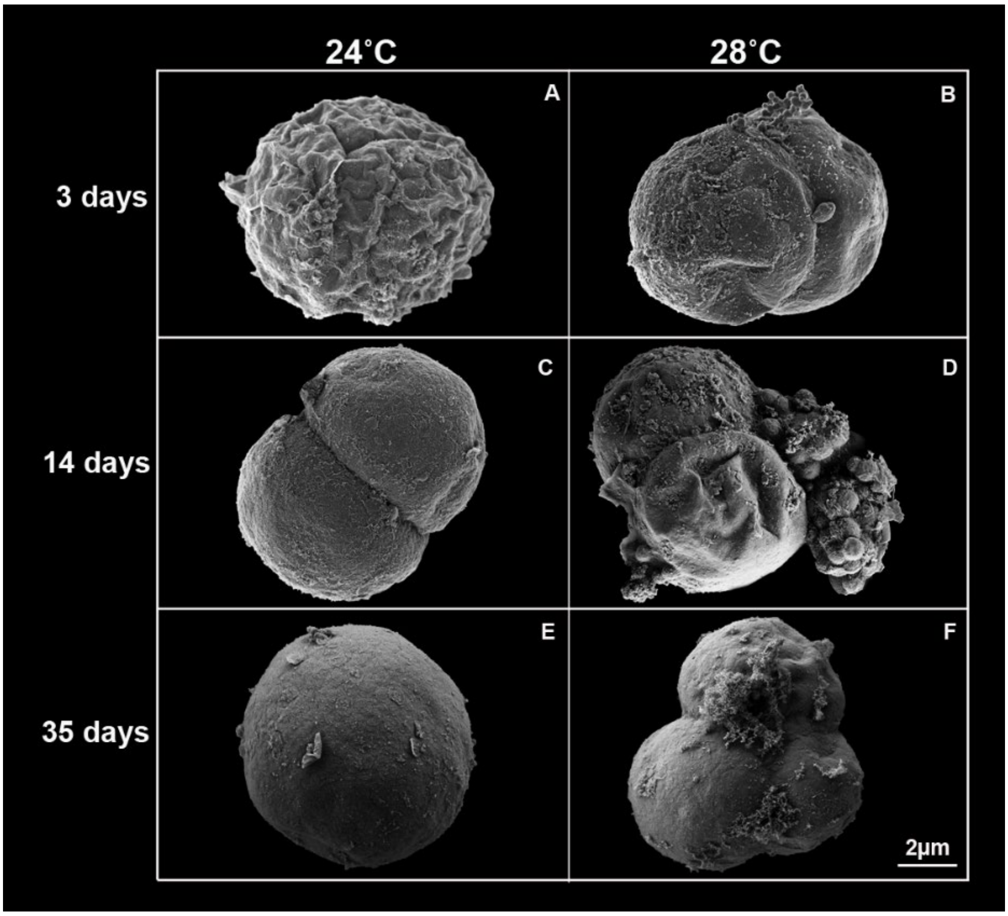

Fig.S2: Scanning electron microscopy showing ultrastructural changes during stress heated at different days in *Cladocopium* cells. *Cladocopium* cells cultured at 24°C (Panels A-C and E) and 28°C (Panels B-D and F). First line represent cells collected in 3 days, second line in 14 days and third line in 35 days. The scale bars indicate in images.
